# Correction to “Half‐Pipe Melt Electrowritten Scaffolds Support Engineering of an Immunocompetent Hydrogel‐Embedded Intestine‐on‐a‐Chip”

**DOI:** 10.1002/advs.77108

**Published:** 2026-08-13

R. Janssen, H. S. Schulze, C. M. L. Nelissen, et al. “Half‐Pipe Melt Electrowritten Scaffolds Support Engineering of an Immunocompetent Hydrogel‐Embedded Intestine‐on‐a‐Chip.” *Advanced Science* 12, no. 37 (2025): 12, e07132. https://doi.org/10.1002/advs.202507132

We identified an error confined to one specific dataset within the paper, namely the immune cell dataset (IEC/moDC, moDC/T cell co‑culture data). Within this dataset, a small number of panels (listed below) show data bars for the IoC and TW conditions that were inadvertently switched during figure preparation. This error affects only these specific panels and does not extend to the remaining figures within this dataset, nor to any other data or sections of the manuscript. The underlying data, statistical analyses, results text, and conclusions of the manuscript, including for this immune cell dataset, are entirely unaffected and remain unchanged. We have re‑generated the affected figures with the correct IoC/TW assignment:


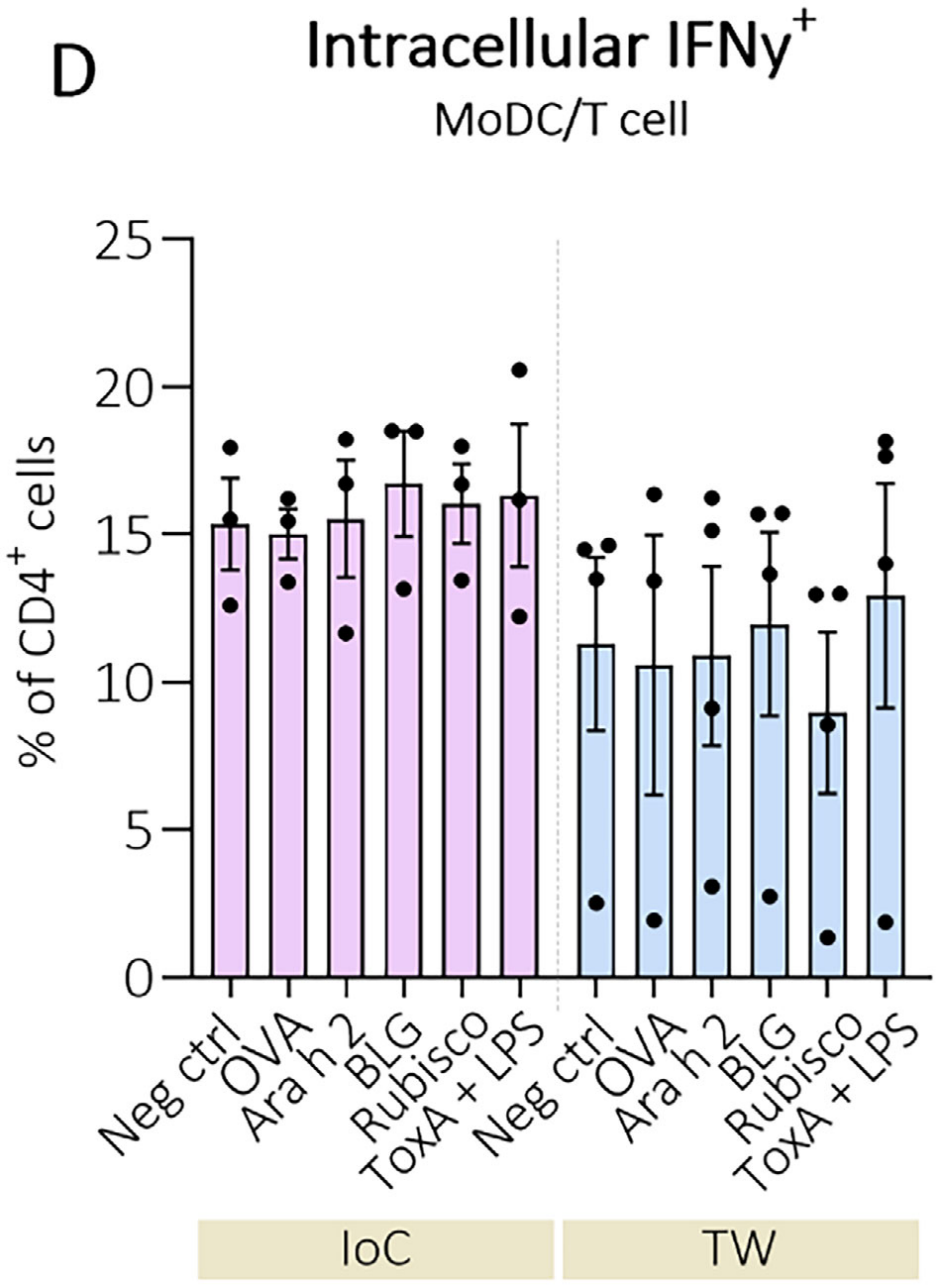


Figure 7D. Display error of IoC and TW conditions.

Corrected Figure 7D.


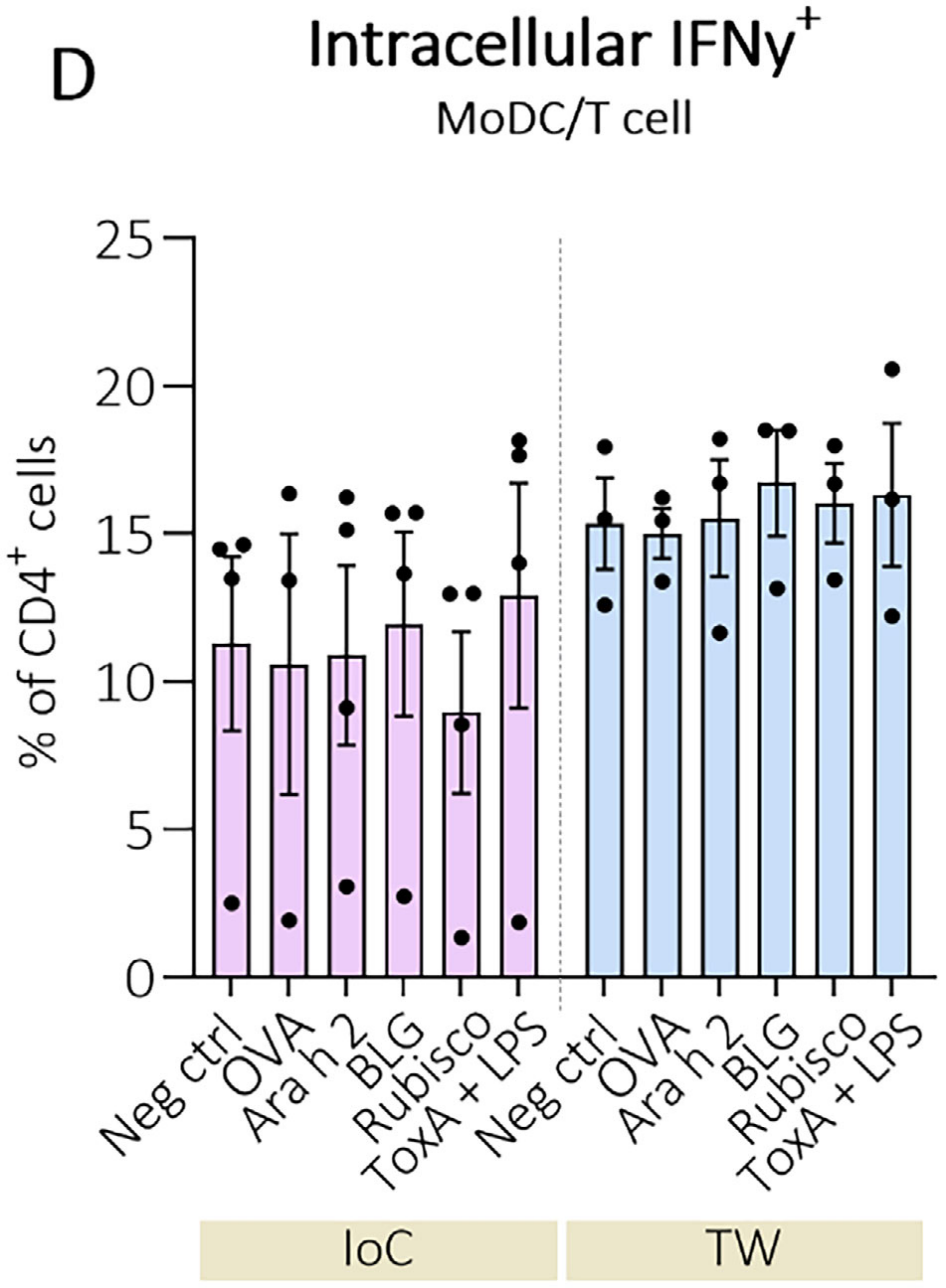


Supplementary figures in Supporting Information:


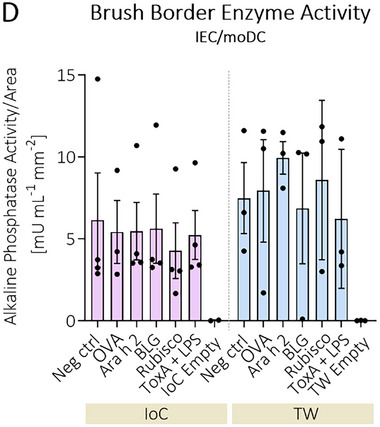


Figure S14D. Display error Rubisco and ToxA + LPS (TW).

Corrected Figure S14D:


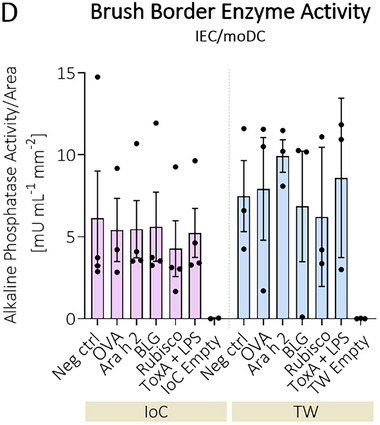



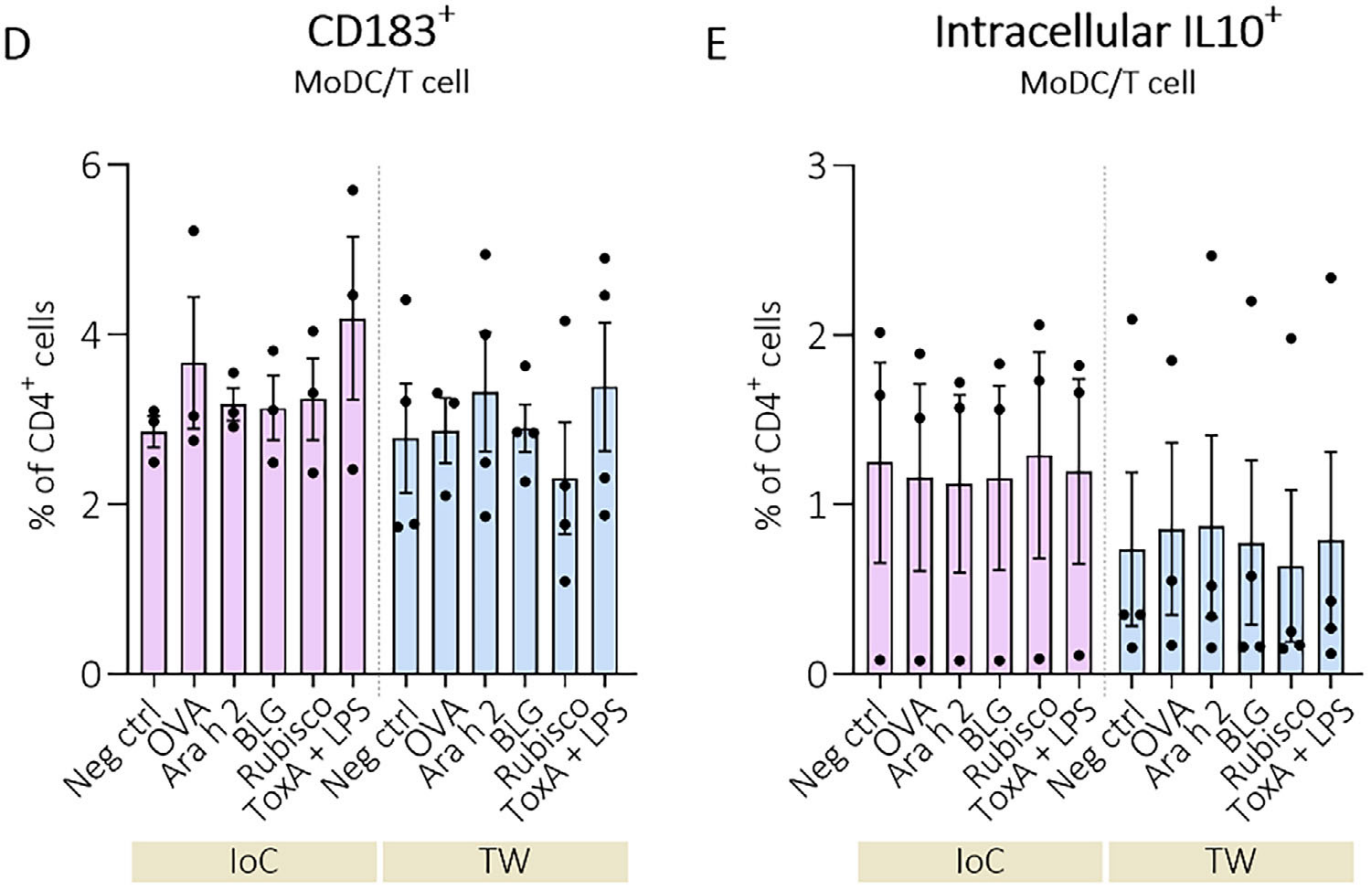


Figure S18D and E. Display error of IoC and TW conditions.

Corrected Figure S18D,E:


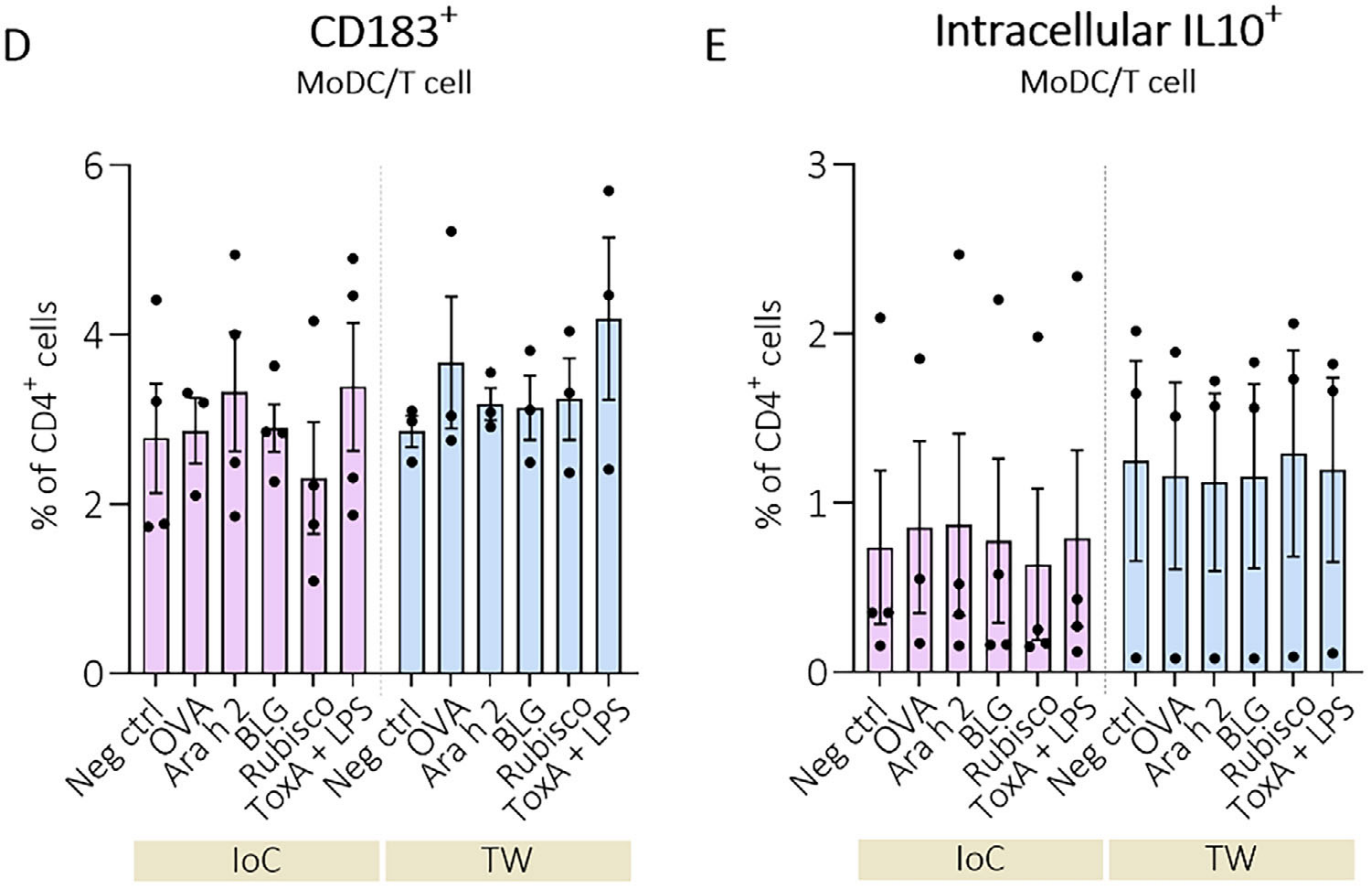


Further, in Equation S1, *C*_0_ is the apical initial concentration should read (nmol mL^−^^1^) instead of (nmol L^−^^1^). It does not affect any results, statistical analyses, or conclusions presented in the article.

We sincerely apologize for these errors.

